# Novel Insights into Total Flavonoids of *Rhizoma Drynariae* against Meat Quality Deterioration Caused by Dietary Aflatoxin B1 Exposure in Chickens

**DOI:** 10.3390/antiox12010083

**Published:** 2022-12-30

**Authors:** Ke Yue, Kai-Li Liu, Yao-Di Zhu, Wen-Li Ding, Bo-Wen Xu, Aftab Shaukat, Yan-Feng He, Lu-Xi Lin, Cai Zhang, Shu-Cheng Huang

**Affiliations:** 1 Department of Clinical Veterinary Medicine, College of Veterinary Medicine, Henan Agricultural University, Zhengzhou 450046, China; 2 College of Food Science and Technology, Henan Agricultural University, Zhengzhou 450002, China; 3 National Center for International Research on Animal Genetics, Breeding and Reproduction (NCIRAGBR), Huazhong Agricultural University, Wuhan 430070, China; 4 Laboratory of Environment and Livestock Products, Henan University of Science and Technology, Luoyang 471023, China

**Keywords:** aflatoxin B1, metabolomics, meat quality, oxidative stress, Raman spectroscopy, texture profile analysis

## Abstract

Aflatoxin B1 (AFB1) is a group of highly toxic mycotoxins that are commonly found in human and animal foods and threaten animal and human food safety. Total flavonoids of *Rhizoma Drynaria* (TFRD), a traditional Chinese medicinal herb, exert multiple biological activities such as immunomodulatory, anti-inflammatory, and anti-oxidation effects. Here, a total of 160 healthy 21-day-old male broilers were randomly divided into four groups: the CON group, the TFRD group, the AFB1 group, and the AFB1 + TFRD group. The study found that AFB1 exposure altered the breast meat quality-related indicators, including meat sensory and physical indicators. Metabolomics analysis further showed that the change in meat quality was closely associated with significantly differential metabolites of breast muscle. Furthermore, spotlighted amino acid content contributes to changes in the secondary structure of the myofibrillar protein by Raman spectroscopy analysis, which was associated with the oxidative stress and inflammatory response in AFB1-exposed breast meat. Meanwhile, dietary 125 mg/kg TFRD supplementation could effectively restore the changes in breast meat quality. Taken together, these results by multi-technical analysis revealed that AFB1 exposure causes deterioration of chicken meat quality and that TFRD may be a potential herbal extract to antagonize mycotoxicity.

## 1. Introduction

The Food and Agriculture Organization of the United Nations (FAO) survey shows that nearly a quarter of the world’s cereal crops are threatened by mycotoxins, such as wheat, corn, soybeans, silage, and haylage [1,2]. Aflatoxin B1 (AFB1) is the most abundant and toxic of the aflatoxins (AFs), which are secondary metabolites of fungi of the genus *Aspergillus*, and is much more toxic than cyanide, heavy metals, and organic pesticides [3]. AFB1 is most likely to be produced during the production, storage and transportation of agricultural products and is extremely widespread, while animals are further threatened by the consumption of AFB1-contaminated agricultural products [4]. Previous studies have focused on the health risks of AFB1 to animals, ignoring the threat it poses to humans through the contamination of animal-derived foods [5]. Broiler breast meat is an important source of protein from animal-derived foods origin for humans, which has become a significant consumer product in many countries due to its low fat, high protein, and low price [6]. However, in the standardized broiler feeding process, broiler feed is at high risk of mycotoxin, which not only affects the health of the broiler itself but may also threaten the quality of the broiler breast meat [7,8].

Previous studies showed that the most perceptible and important meat product characteristics, including tenderness, juiciness, flavor, and appropriate texture, which sway consumers’ initial and final quality judgment during meat product purchases [9]. Texture profile analysis is a double compression cycle that assesses multiple meat quality variables, including hardness, springiness, cohesiveness, gumminess, chewiness, and resilience, which can be used as indicators for the sensory assessment of meat quality [10]. Moreover, these indicators are closely related and have mutual influence, and are also essential for processors involved in the value-added production of meat products [9]. Among them, WHC, including dripping loss and cooking loss, is one of the essential functional characteristics of raw meat, which is directly related to the color and tenderness of meat [9]. The WHC was also shown to be closely associated with T_21_ and T_22_ in the nuclear magnetic resonance (NMR) transverse relaxation (T_2_) measurements [11]. We can evaluate the toxic effects of AFB1 on broiler breast muscle quality from a variety of perspectives using the above methods.

The total flavonoids of *Rhizoma drynariae* (TFRD) are derived from the main components of the dried rhizome of *Rhizoma drynariae*, a traditional Chinese medicine, that has many pharmacological activities such as immunomodulatory, anti-inflammatory, and anti-oxidation effects [12,13]. Previous studies have demonstrated that AFB1 causes target organ dysfunction mainly by causing oxidative stress and inflammatory responses [14]. Interestingly, TFRD was shown to improve the osteoporosis model in mice by reducing oxidative stress and anti-inflammatory effects in a related study [13]. Moreover, the scavenging ability of TFRD on hydroxyl radical and peroxy anion-free radical was 10.12% and 35.71%, respectively, and there was an apparent dose–effect relationship between the mass concentration of flavonoids, suggesting that TFRD can be used as a natural antioxidant [15]. In this study, we found for the first time that TFRD, a natural antioxidant, could improve the deterioration of broiler breast meat quality caused by AFB1 by inhibiting inflammatory factors and increasing antioxidant enzyme activity, and more importantly, alleviate the metabolic disorders and protein secondary structure changes in muscle fibers in breast muscle

Therefore, this study further explored whether TFRD as a feed additive could alleviate the deterioration of breast muscle meat quality caused by AFB1 exposure by examining sensory and physical changes in breast muscle, changes in breast muscle metabolite levels, changes in protein secondary structure of myofibrillar, and assessment of inflammatory responses and oxidative damage in breast muscle.

## 2. Materials and Methods

### 2.1. Animal Ethics

All animal experiment procedures were conducted following the guidelines of the Animal Welfare and Ethics Research Committee, College of Veterinary Medicine, Henan Agricultural University (ethical approval code: 170126) (Zhengzhou, China). No signs of pain or suffering we detected in all broiler testing procedures.

### 2.2. Animal Experiment

**Figure 1:**
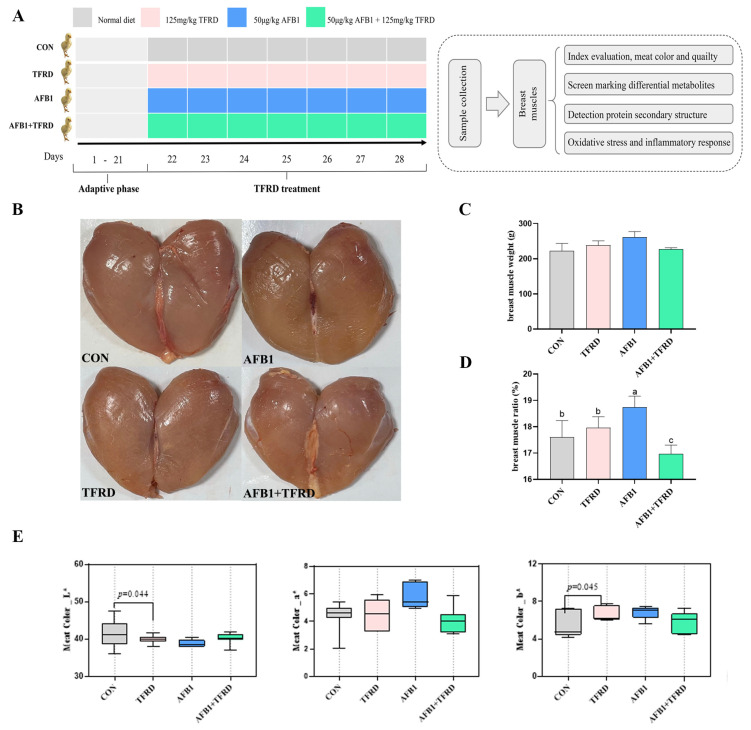
Effect of TFRD on AFB1-induced breast muscle color parameters in broilers. (**A**) Schematic diagram of experimental design to study the effect of TFRD on AFB1-induced breast muscle injury in broilers. (**B**) Representative color images of breast muscle in the CON, TFRD, AFB1, and AFB1 + TFRD groups were observed during the entire experimental period. (**C**,**D**) Relative changes in breast muscle and breast muscle ratios were recorded and analyzed over the entire experimental period. (**E**) The color [lightness (L*), redness (a*), and yellowness (b*)] of the breast muscles in the CON, TFRD, AFB1, and AFB1 + TFRD groups were measured and analyzed. Different letters indicate statistically significant differences (*p* < 0.05), and all values are presented as the means ± SD, one-way ANOVA.

Three weeks-old arbor acres (AA) broilers (body weight, 838.14 ± 5.64 g) were purchased from the XingDa Poultry Industry Co., Ltd. (Kaifeng, China). 160 healthy broilers were randomly arranged into four groups with four replicate cages and 10 chicks in each cage, namely, CON group (normal diet), TFRD group (basal diet mixed with 125 mg/kg TFRD (#K20798, Xi’an Kailai Biological Engineering Co., Ltd., Xi’an, China), AFB1 group (50 μg/kg body weight AFB1 (#C2J0G2, Qingdao Pribolab Biological Engineering Co., Ltd., Qingdao, China), and the AFB1 + TFRD group (Figure 1A). The reference doses and usage of TFRD and AFB1 were similar to those described previously, with TFRD at 125 mg/kg (diet) well mixed in the broiler basal diet and AFB1 dissolved in ethanol solution and injected in the broiler crop at 50 μg/kg (body weight) [16]. The main components of TFRD determined by HPLC are shown in Table S1. During the entire 7-day experimental cycle, the four groups were in exactly the same rearing environment, given fresh water, ad libitum feeding, and 12:12 h dark or light cycles for broilers, which were kept under conditions and feed standards in accordance with the Chinese Chicken Feeding Standard (MAPRC, 2004. NY/T33-2004).

### 2.3. Sample Collection

Blood samples were collected aseptically from the wing vein using heparinized syringes in each group after 7 days of continuous experimental intervention. The plasma was separated by centrifugation at 3000× *g* rpm (4 °C) for 10 min, then plasma was directly aliquoted and stored at −20 °C. Ten broilers in each group were sacrificed with dislocation at 28 days of age (slaughter weight 1329.61 ± 22.75 g), and broiler breast muscle was weighed by using a 0.001 g sensitive electronic balance. Next, the collected breast muscle samples were randomly divided into two parts, one part for meat quality evaluation and detection of antioxidant-related genes and the other for metabonomics sequencing analysis (Figure 1A).

### 2.4. Analysis of Meat Color and of the Breast Muscle

During the evaluation of the chicken meat color, the full view of the breast muscle was first observed, photographed, and recorded (*n* = 10). At 45 min postmortem, the meat color parameters of intact skinless breast muscle including lightness (L*), yellowness (b*), and redness (a*) were measured using a tristimulus colorimeter (#SR-62, 3nh, Shenzhen, China) according to the CIELAB system. Meanwhile, all pH values of the breast muscle samples were measured by using a precision pH meter (#testo206, Tetu, Shanghai, China). Each breast muscle sample was measured 3 times at three different locations and the average of the readings was taken as the meat color and pH value as described in our previous study [17].

### 2.5. Analysis of Water-Holding Capacity (WHC) of the Breast Muscle

In meat quality evaluation, drip loss and cooking loss are recognized parameters to evaluate the WHC of meat. In this experiment, drip loss and cooking loss of breast muscle samples were measured at 24 h postmortem. The procedure was performed as described previously [17], and used the following equation for calculation: drip loss (%) or cooking loss (%) = [(initial weight−final weight)/initial weight] × 100. Each group with ten breast muscle samples was measured and calculated to assess WHC.

### 2.6. Analysis of Shear Force of the Breast Muscle

Chicken shear force was determined by using a digital display muscle tenderness meter (#C-LM36, Northeast Agricultural University School of Engineering, Harbin, China) as described in our previous study [17]. Each breast muscle sample was cut 3 times and the average of the readings was taken as the shear force value.

### 2.7. Texture Profile Analysis (TPA) Determination

The breast muscle sample was heated in a water bath for 15 min, then trimmed into a square of 1 cm × 1 cm × 1 cm. TA.XTplus type physical property tester was used for measurement, and the texture profile was analyzed in “secondary compression” mode. The first compression pre-test rate was 20 mm/s, the test rate was 15 mm/s, and the post-test rate was 10 mm/s with a compression degree of 60%. The second compression height of rising was 30 mm, the pre-test rate was 20 mm/s, the test rate was 2 mm/s, the post-test rate was 100 mm/s, and the compression degree was 40% [18]. Data were collected and analyzed using Texture Exponent 32 software (Supertech Instruments Ltd., Beijing, China). The final breast TPA results were expressed in terms of hardness, chewiness, gumminess, springiness, cohesiveness, and resilience.

### 2.8. LF-NMR Transverse Relaxation

The breast muscle samples were trimmed to a rectangular shape (1 × 1 × 2 cm, about 2 ± 0.1 g) after being heated for 15 min and were put into glass tubes. The distribution and mobility of breast muscle water were measured by using the low-field nuclear magnetic resonance (LF-NMR) transverse relaxation technique with a Niumag Pulsed nuclear magnetic resonance (NMR) analyzer (#PO001, Niumag Co., Ltd., Shanghai, China), and detected with a proton resonance frequency of 22 MHz at 25 °C. Next, the transverse relaxation times (T_2_) measurement, pulse length, and repetition time between two scans are consistent with those previously reported [19]. Analysis of breast muscle echoes data using the MultiExp Inv Analysis program version 4.08 (Niumag Co., Ltd., Shanghai, China).

### 2.9. Analysis of Raman Spectroscopy of the Breast Muscle

The breast muscle samples were used for Raman analysis to analyze the protein secondary structure using a confocal Raman micro-spectrometer (LabRAM HR Evolution, Horiba France SAS, Villeneuve-d’Ascq, France). Before sample detection, the 520.7 cm^−1^ peaks were used as the reference peak for the calibration of the Raman instrument using silicon wafers (Si). Surface-enhanced Raman spectra were acquired using a 532.8 nm laser as the excitation light source. The laser intensity was set to the maximum intensity (50 mw), the objective set to 50 ×, and the integration time 30 s for three times. The detected spectra were from 400 to 1800 cm^−^^1^ with a resolution of 1 cm^−1^. The prepared and processed samples were tested on the machine, and 12 random acquisitions were made for each sample separately. Next, the normalization of spectra, multi-point baseline correction, and background treatment are consistent with those previously reported [20]. Table S2 displays the tentative assignments of the main bands in the Raman spectra [20]. The secondary structure of proteins (including α-helix, β-sheet, β-turn, and random coil) was determined according to the method described by Susi and Byler [21] and was presented using an online tool. (https://www.genescloud.cn/chart/ChordPlot, accessed on 1 February 2021).

### 2.10. Determination of Antioxidant Enzyme Activity in Plasma

Six plasma samples were randomly taken from each group for the determination of the activities of related antioxidant enzymes in plasma including the concentration of catalase (CAT), total superoxide dismutase (T-SOD), glutathione peroxidase (GSH-Px), reduced glutathione (GSH), and malondialdehyde (MDA), according to the manufacturer’s instructions, measured by kits from Nanjing Jiancheng Bioengineering Co., Ltd. (Jiangsu, China).

### 2.11. RNA Extraction and qRT- PCR Analysis 

Total mRNA was isolated from liquid nitrogen-frozen breast muscle samples using 1000 μL TRIzol reagent (China Bioengineering Co., Ltd., Beijing, China). Before reverse transcription (RT), the concentration of RNA was quantified by Nanodrop 2000 (Thermo Fisher Scientific Co., Ltd., Beijing, China) spectrophotometer. Primers specific for oxidation-related genes (*GST*, *GPX*, *CAT*, and *SOD*), and Inflammation-related genes (*IL-1β*, *IL-6*, *IL-10*, and *TNF-ɑ*), and GAPDH were designed by Primer Premier 5.0 software (Table S3) and synthesized Tsingke Biological Technology Co., Ltd. (Wuhan, China). GAPDH was used as an internal control gene. The 2^−ΔΔCt^ approach was used to calculate the relative abundance of the targeted genes. Targeted gene mRNA expression in CON was used as baseline relative to groups.

### 2.12. Breast Muscle Metabolite Analysis by Widely Targeted Metabolomics

For metabonomic analysis, approximately 50 mg of breast muscle samples was collected from the CON, AFB1, and AFB1 + TFRD group broiler chickens. Each breast muscle sample and its quality control (QC) was prepared and performed to follow a previously reported procedure before UPLC-MS/MS analysis [17]. All sequencing data analysis of breast muscle samples was based on the UPLC-MS/MS detection platform and a self-built MWDB database (Metware Biotechnology Co., Ltd. Wuhan, China). 

The identified metabolite data were further normalized by log2-transformation for Orthogonal Partial Least Squares-Discriminant Analysis (OPLS-DA), which also contained score plots and permutation plots of OPLS-DA, generated using the R package MetaboAnalystR. Significantly differential metabolites between groups were detected by variable important in projection (VIP)  >  1 and fold change (FC) ≥ 2 or FC ≤ 0.5. Next, identified metabolites were annotated and then mapped to the Kyoto Encyclopedia of Genes and Genomes (KEGG) pathway database (http://www.kegg.jp/kegg/pathway.html, accessed on 1 February 2021). In addition, Circos, Sankey, and ternary plots of the identified metabolites were presented using an online tool. (https://www.genescloud.cn/chart/ChordPlot; https://www.genescloud.cn/chart/TraceSankeyPlot; https://www.omicshare.com/tools/Home/Soft/ternary_plot, accessed on 1 February 2021).

### 2.13. Statistical Analysis

One-way ANOVA for the multiple-groups comparisons or Student’s *t*-test for two-group comparisons were performed on all experimental data using GraphPad Prism version 8.0.0 for Windows (GraphPad Software, La Jolla, CA, USA). The data are presented as the mean ± standard deviation. In addition, Pearson’s analysis was performed to analyze the relationship between meat quality-related indicators, breast muscle differential metabolites, and plasma oxidative damage and inflammation-related indicators. In all of our analyzed data, *p* < 0.05 were considered statistically significant.

## 3. Results 

### 3.1. Color Evaluation of Breast Muscle on

As shown in Figure 1B, color observations showed that the bright redness of broiler breast muscle in the AFB1 group was lower than that of the CON group. Moreover, the breast muscle color of broilers was further assessed by using a high-quality colorimeter, which showed that the TFRD group had a considerable reduction in L* values (*p* = 0.044), while a significant increase (*p* = 0.045) in b* values compared to the CON group (Figure 1E). It is noted that no significant differences in broiler breast muscle color index L* values (*p* = 0.303), a* values (*p* = 0.816), and b* values (*p* = 0.817) between the AFB1 + TFRD and CON groups (Figure 1E). Additionally, measurements of broiler breast muscle weights showed no significant difference between the groups (Figure 1C), but a significant increase in breast muscle ratio in the AFB1 group compared to the CON group (*p* = 0.046), while a markedly decrease in the AFB1 + TFRD group compared to the AFB1 group (*p* = 0.013) (Figure 1D). These results indicated that TFRD treatment could protect against AFB1-induced changes in breast muscle appearance and breast muscle status of broiler chickens.

### 3.2. Meat Quality Assessment

**Figure 2:**
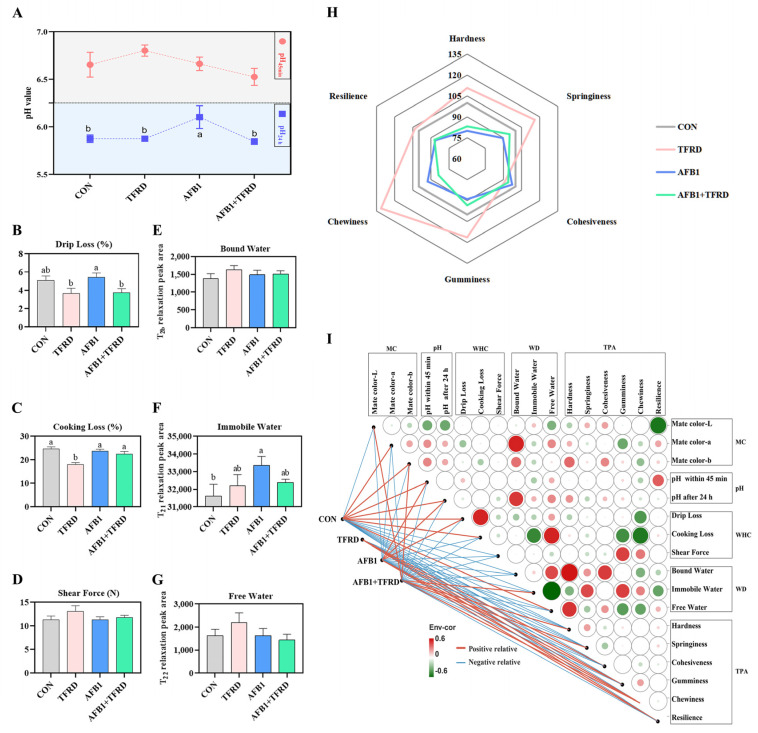
Effect of TFRD on AFB1−induced meat quality of the breast muscle in broilers. (**A**) Comparison of pH value of the breast muscle in the CON, TFRD, AFB1, and AFB1 + TFRD groups during the entire experimental period. (**B**–**D**) Comparison drip loss, cooking loss, and shear force of the breast muscle in the CON, TFRD, AFB1, and AFB1 + TFRD groups. (**E**–**G**) Comparison of water distribution including bound water, immobile water, and free water of the breast muscle in the CON, TFRD, AFB1, and AFB1 + TFRD groups. (**H**) Comparison of texture profile analysis including hardness, springiness, cohesiveness, gumminess, chewiness, and resilience of the breast muscle in the CON, TFRD, AFB1, and AFB1 + TFRD groups. (**I**) Pearson correlation analysis between meat quality assessment indicators. Note: The different rectangles are colored based on the Pearson correlation coefficients between meat quality assessment indicators. The intensity of color represents the degree of correlation, green represents positive correlation, red represents negative correlation. The edges indicate the correlation between breast muscle ratios and the meat quality assessment index, red for the positive correlation and blue for the negative correlation. MC: meta color, WHC: water-holding capacity, WD: water distribution, TPA: texture profile analysis. Different letters indicate statistically significant differences (*p* < 0.05), and all values are presented as the means ± SD, one−way ANOVA.

AFB1 affects the appearance and condition of broiler breast muscle and also causes deterioration of breast muscle meat quality. The meat quality evaluation was carried out in 6 terms, including pH value, drip loss, cooking loss, shear force, water dynamic distribution, and texture characteristics of the breast muscle. First, there was no significant change in the pH values of the breast muscle of the four experimental groups within 45 min of slaughter. Twenty-four hours later, the pH values of the breast muscle of the AFB1 group were dramatically higher than those of the CON group (*p* < 0.05); the TFRD-treated group significantly inhibited the increase in pH values caused by AFB1 exposure, as illustrated in Figure 2A (*p* < 0.05). Compared with the CON group, the AFB1 group showed an increasing trend in drip loss (%) in the breast muscle, but the difference was not significant (*p* = 0.629). However, the drip loss in the breast muscle was significantly reduced in the AFB1 + TFRD group compared to the AFB1 group (*p* = 0.022; Figure 2B). In addition, the TFRD group showed a prominent decrease (*p* < 0.05) in cooking loss of breast muscle compared to the other three groups (Figure 2C), but the shear force in this study was not significantly different between the groups (Figure 2D). 

NMR transverse relaxometry (T_2_) using LF-NMR characterized the myofibrillar water distribution and mobility as well as structural features in meat, and water distribution (WD) and mobility are important for the meat quality [22]. Next, to further explore whether AFB1-induced changes in drip loss are related to the dynamic water distribution of breast muscle, the bound water (T_2b_), immobile water (T_21_), and free water (T_22_) of breast muscle were analyzed by using NMR transverse relaxation (T_2_) measurements. The results showed no significant change inbound water and free water of breast muscle among the four groups (Figure 2E,G). However, there were dramatic differences in the amount of immobile water most abundant in the four breast muscle groups. There was a significant increase in immobile water in the breast muscle in the AFB1 group compared to the CON group (*p* = 0.041). In contrast, there was no significant difference in immobile water in the TFRD and AFB1 + TFRD groups (*p* = 0.437 and 0.319, respectively).

The quality of broiler breast muscle was assessed not only in terms of physical properties including pH, WHC, and WD of the meat, but also the sensory evaluation of the breast muscle by TPA. The results showed that compared with the CON group, the TPA indicators in the AFB1 group showed a decreasing trend of hardness, springiness, gumminess, chewiness, and resilience in the breast muscle (*p* = 0.011, 0.135, 0.291, 0.585, and 0.009, respectively). On the contrary, these TPA indicators were significantly increased in the AFB1 + TFRD group compared with the AFB1 group (*p* = 0.001, 0.001, 0.201, 0.013, 0.002, and 0.003, respectively; Figure 2H). Pearson correlation analysis between meat quality assessment indicators and the edges indicated the correlation between breast muscle ratios and the meat quality assessment index (Figure 2I). The L* of breast muscle flesh color was negatively correlated with resilience (r = −0.543; *p* = 0.003). The drip loss showed a significant negative correlation with chewiness (r = −0.418; *p* = 0.027). The cooking loss showed a significant positive correlation with drip loss and free water (r = 0.517, and 0.519; *p* = 0.005, and 0.023), showed a significant negative correlation with immobile water, gumminess, and chewiness (r = −0.476, −0.450, and −0.530; *p* = 0.039, 0.016, and 0.04). The bound water showed a significant positive correlation with the a* of breast muscle flesh color, pH value after 24 h, hardness, and Cohesiveness (r = 0.522, 0.470, 0.555, and 0.462; *p* = 0.022, 0.042, 0.014, and 0.047). 

These results collectively demonstrated that AFB1 exposure might affect the normal acid discharge process and change in breast muscle pH, thus causing a reduction in water retention ability of breast muscle and altering texture characteristics of breast muscle. Meanwhile, the TFRD treatment can protect against AFB1-induced damage in breast muscle meat quality of broiler chickens.

### 3.3. Analysis of Breast Muscle Metabolic Profiles after TFRD Treatment in AFB1-Exposed Chickens

**Figure 3:**
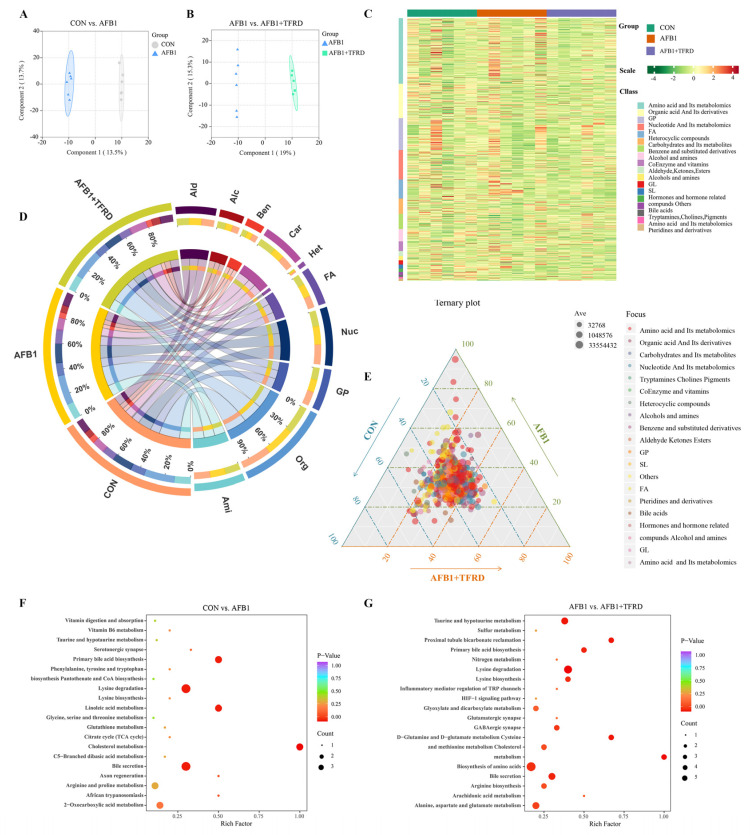
Analysis of breast muscle metabolism after TFRD treatment in AFB1-exposed chickens. (**A**,**B**) OPLS-DA analysis of LC-MS/MS of broiler breast muscles, CON vs. AFB1 and AFB1 vs. AFB1 + TFRD. (**C**) Cluster analysis of LC-MS/MS of broiler breast muscles. (**D**) Metabolite abundance analysis of LC-MS/MS of broiler breast muscles. Ami, amino acid and its metabolomics; Org, organic acid and its metabolomics; GP, glycerol phospholipids; Nuc, nucleotide and its metabolomics; FA, fatty acid; Het, heterocyclic compounds; Car, carbohydrates and its metabolomics; Ben, benzene and substituted; Alc, alcohol and amines; Ald: aldehyde, ketones, esters. (**E**) Ternary analysis of LC-MS/MS of broiler breast muscles. The degree of concentration of the points indicates that the metabolites are evenly distributed among the three groups. The degree of dispersion of the points indicates that the metabolites differ significantly among the three groups. (**F**,**G**) Potential metabolic pathway analysis based on significantly differential metabolites in breast muscles from the CON, AFB1, and AFB1 + TFRD group broiler chickens. The degree of enrichment was analyzed by a rich factor, *p*-value, and the number of metabolites that were enriched in each pathway. The size of the bubble indicates the number of significant differential metabolites that are enriched in this pathway, and the point with the different gradation of color (from red to blue) represents the scope of the *p*-value. The larger size of each circle indicates a higher degree of enrichment, and the lower *p*-value represents a more significant degree of enrichment.

AFB1 causes changes in breast muscle meat quality caused by disruption of metabolites within the breast muscle. Analysis of metabolism difference in broiler breast muscle samples by UPLC-MS/MS metabolomics sequencing. A total of 785 metabolites were detected in the CON group, AFB1 group, and AFB1 + TFRD group. The supervised OPLS-DA method was performed to assess the breast muscle samples and the OPLS-DA score plots of CON vs. AFB1 group and AFB1 vs. AFB1 + TFRD group showed a significant separation, indicating differences in the metabolic profiles of each two groups (Figure 3A,B). Differential metabolites were screened for the comparison group by combining the criteria of |log2(FC)| ≥ 1 and VIP value > 1 of the metabolites. The results presented the metabolites classification of breast muscle in the CON group, AFB1 group, and AFB1 + TFRD group including amino acid and its metabolites, organic acid and its derivatives, glycerol phospholipids (GP), nucleotide and its metabolites, fatty acids (FA), heterocyclic compounds, etc., and the classification with the highest number of metabolites is amino acids and its metabolites, followed by organic acid and its derivatives by using a cluster analysis as illustrated in heap map (Figure 3C).

A Circos plot of the metabolites classification of breast muscle in different groups was constructed to show the proportion of the metabolites in each group. The abundance of metabolite classifications is counted and analyzed (Figure 3D). The results showed that the proportion of fatty acids in the AFB1 group (36.88%) was greater than that in the CON and AFB1 + TFRD groups (32.42% and 30.69%, respectively). Conversely, the proportion of carbohydrates and their metabolites in the CON and AFB1 + TFRD groups (30.04% and 42.06%, respectively) was greater than that in the AFB1 group (28.89%). In addition, the inconsistencies in the number and abundance of metabolite classifications were noteworthy. Amino acids and their metabolomics are quantitatively the most abundant metabolite classification, but the abundance proportion of the organic acids and their metabolomics was greater than amino acids and their metabolomics. Heterocyclic compounds have more quantities of metabolites than carbohydrates and their metabolomics, but the abundance proportion of carbohydrates and their metabolomics was greater than heterocyclic compounds. The ternary plot analysis of metabolites is shown in Figure 3E and is plotted according to the different proportions of the same metabolite in the three groups. The results showed that metabolites in the classification of amino acid and its metabolomics were higher in the AFB1 group than in the CON and AFB1 + TFRD groups. The above results suggested that AFB1 exposure impairs the meat quality of breast muscle by affecting amino acids, organic acids, carbohydrates, fatty acids, and its metabolites in breast muscle.

Moreover, the results of KEGG enrichment analyses of the significantly different metabolites between the experimental groups showed that broiler chickens with AFB1 mainly enriched in primary bile acid biosynthesis, lysine degradation, linoleic acid metabolism, cholesterol metabolism, and bile secretion (Figure 3F). In addition, broilers supplemented with TFRD mainly enriched in amino acid metabolism (lysine degradation, lysine biosynthesis, biosynthesis of amino acids, arginine biosynthesis, D-glutamine, and D-glutamate metabolism and alanine, aspartate, and glutamate metabolism), energy metabolism (glyoxylate and dicarboxylate metabolism), and lipid metabolism (taurine and hypotaurine metabolism, primary bile acid biosynthesis, cholesterol metabolism, and bile secretion) (Figure 3G). Based on the above results, AFB1 exposure affects the meat quality mainly by altering lysine degradation, cholesterol metabolism, and bile secretion in broiler breast muscle. Moreover, TFRD treatment prevented AFB1-induced changes in breast muscle quality in broilers mainly by improving amino acid metabolism, energy metabolism, and lipid metabolism.

### 3.4. Metabolomics Screening of Significantly Different Metabolites in Breast Muscles of AFB1 Chickens after TFRD Treatment

**Figure 4:**
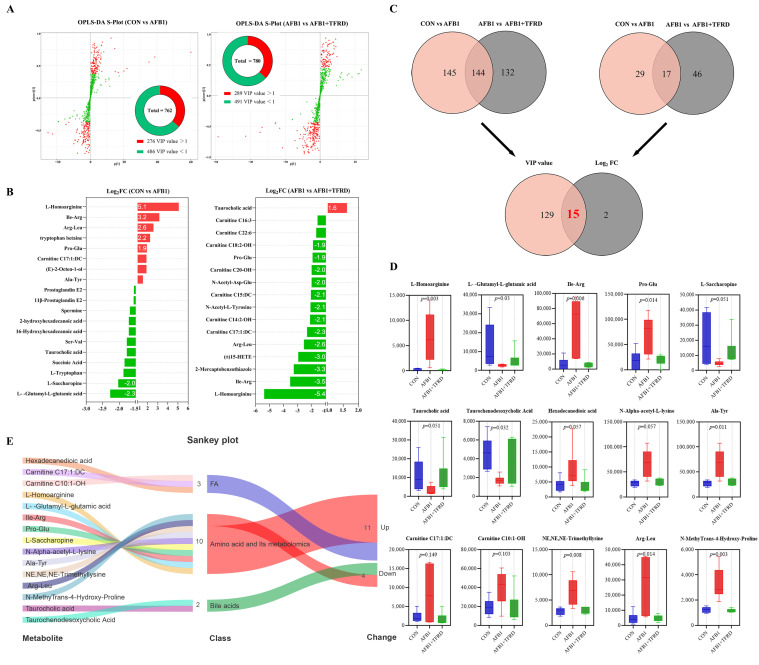
Screening of significantly differential metabolites in breast muscles after TFRD treatment in AFB1-exposed chickens by metabolomics analysis. (**A**) S-plot of OPLS-DA analysis of breast muscle metabolites. Significantly differential metabolites (VIP > 1) are noted as a red dot, whereas a green dot represents no significant difference in metabolites. The number of metabolites that meet the criteria (VIP > 1) is shown in a pie chart. (**B**) Visualization of log2-transformed read counts of differential metabolites in breast muscles between the CON vs. AFB1 and AFB1 vs. AFB1 + TFRD groups (|log2FC| ≥ ±1.5, FC stands for fold-change). (**C**) Venn analysis was performed by |log2FC| ≥ ±1 and VIP > 1 to screen for differential breast muscle metabolites. The overlap of the Venn diagram represents the number of differential breast muscle metabolites common to the different comparison groups. (**D**) The overlap of VIP values and log2(FC) in the Venn diagram indicates the number of differential metabolites shared between groups. Statistical analysis was performed for metabolites that differed between groups; *p* < 0.05 indicates statistical significance between the two variables, and all values are expressed as mean ± SD with one-way ANOVA. (**E**) Sankey plots depict the class and trends of marker differential metabolites in the AFB1 group of breast muscles.

An S-plot for OPLS-DA for the two comparison groups is a graphical representation of the VIP within the OPLS-DA model, with the statistics contained within the S-plot for OPLS-DA presented in the form of a pie chart (Figure 4A). The results showed 762 metabolites in the CON vs. AFB1 group, including 276 metabolites with VIP values > 1. There are 780 metabolites in the AFB1 vs. AFB1 + TFRD group, including 289 metabolites with VIP values > 1. The metabolites with differences in breast muscle metabolites were screened by difference analysis, and log2FC represented the difference score of the two groups of metabolites (Figure 4B). The comparison between the AFB1 group and the CON group showed that L-homoarginine (log2FC = 5.1, *p* = 0.031), Ile-Arg (log2FC = 3.2, *p* = 0.015), arg-leu (log2FC = 2.6, *p* = 0.025), and tryptophan betaine (log2FC = 2.2, *p* = 0.002) were more abundant, while L-glutamyl-L-glutamic acid (log2FC = −2.3, *p* = 0.098), and L-saccharopine (log2FC = −2.0, *p* = 0.072) were less abundant. Interestingly, compared with the AFB1 group, AFB1 + TFRD group showed that L-homoarginine (log2FC = −5.4, *p* = 0.031), ile-arg (log2FC = −3.5, *p* = 0.015), 2-mercaptobenzothiazole (log2FC = −3.3, *p* = 0.045), (±)15-HETE (log2FC = −3.0, *p* = 0.073), Arg-Leu (log2FC = −2.6, *p* = 0.025), and carnitine C17:1:DC (log2FC = −2.3, *p* = 0.085) were less abundant, while taurocholic acid (log2FC = 1.6, *p* = 0.175) was more abundant.

To further screen key metabolites between groups as potential biomarkers, VIP value > 1 and |log2(FC)| ≥ 1 as screening conditions, a Venn diagram was used to analyze the differential metabolites that commonly exist and meet the screening conditions in the two comparison groups of CON vs. AFB1 group and AFB1 vs. AFB1 + TFRD group (Figure 4C). The results showed that there were 144 metabolites satisfying VIP > 1 and 17 metabolites satisfying |log2(FC)| ≥ 1 in the two comparison groups of CON vs. AFB1 group and AFB1 vs. AFB1 + TFRD group, and finally, 15 significantly different metabolites were identified (Figure 4D). Compared with the CON group, the AFB1 group showed a prominent increase in L-homoarginine’s relative contents, Ile-Arg, Pro-Glu, N-alpha-acetyl-L-lysine Ala-Tyr, carnitine C17:1:DC, carnitine C10:1-OH, NE,NE,NE-trimethyllysine, Arg-Leu, N-methyTrans-4-hydroxy-proline (*p* < 0.05), and Hexadecanedioic acid. It is noted that the above eleven metabolites decreased in the AFB1 + TFRD group compared to the AFB1 group and showed the same trend as the CON group. On the other hand, all of the other four differential metabolites, namely L-glutamyl-L-glutamic acid, L-saccharopine, taurocholic acid, and taurochenodesoxycholic acid, showed significant decreases in the AFB1 group compared to the CON group (*p* = 0.03, *p* = 0.051, *p* = 0.051, and *p* = 0.032, respectively). Furthermore, the Sankey plots showed that amino acids and their metabolomics accounted for a large proportion of the marker difference metabolites in the AFB1 group, which mainly showed increased change than the CON group. In addition, the AFB1 group showed significantly increased changes in FA compared to the CON group, while the changes in bile acids mainly showed a decrease (Figure 4E). The above results indicated that TFRD treatment could protect against AFB1-induced changes in the significantly different metabolites of breast muscle, which are mostly amino acids and their metabolites.

### 3.5. Integrated Analysis of Significantly Differential Metabolites and Meat Quality-Related Parameters 

**Figure 5:**
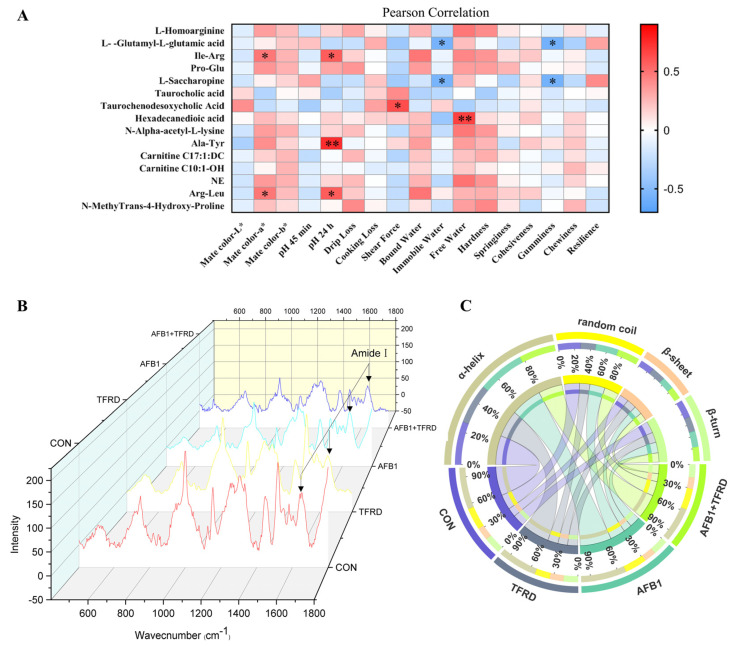
Effects of TFRD on the AFB1-induced protein secondary structure of myofibrillar breast muscle in broilers. (**A**) Pearson correlation analysis between the significantly differential metabolites and indicators of meat quality in breast muscle. Note: The different rectangles are colored based on the Pearson correlation coefficients between the significantly differential metabolites and indicators of meat quality in breast muscle. The intensity of color represents the degree of correlation; blue represents positive correlation, red represents negative correlation. The asterisks indicate statistically significant differences and correspond to *p* < 0.05 (*) and *p* < 0.01 (**). A *p*-value higher than 0.05 represents not statistically significant. (**B**) Raman spectroscopy of breast muscle of broiler chicken at a wavenumber of 400–1800 cm^−1^. (**C**) The abundance analysis of the protein secondary structure of myofibrillar in breast muscle includes ɑ-helix, random coil, β-sheet, and β-turn.

Determination of correlation between breast muscle metabolites and parameters of breast muscle meat quality by Pearson correlation analysis (Figure 5A). Results for meat quality-related indicators depicted that Ile-Arg and Arg-Leu were positively correlated with meat color-a* (r = 0.479 and 0.481, *p* = 0.044 and 0.043, respectively). The pH after 24 h of breast muscle was significantly positively correlated with Ile-Arg, Ala-Tyr, and Arg-Leu (r = 0.573, 0.741, and 0.572, *p* = 0.013, 0.001, and 0.013, respectively). There was a significant positive correlation between shear force and taurochenodesoxycholic acid (r = 0.629, *p* = 0.038). Moreover, L-glutamyl-L-glutamic acid showed a significant negative correlation with immobile water and gumminess (r = −0.564 and −0.578, *p* = 0.029 and 0.012, respectively). Additionally, L-saccharopine demonstrated a significant negative correlation with immobile water and gumminess (r = −0.557 and −0.573, *p* = 0.031 and 0.013, respectively). Hexadecanedioic acid showed a significant positive correlation with free water (r = 0.673 and *p* = 0.006). The results of these correlation analyses clearly indicated that the meat quality index is closely related to the differential metabolites of breast muscle, especially amino acids and their metabolites.

### 3.6. Analysis of the Protein Secondary Structure of Myofibrillar in Breast Muscle after TFRD Treatment in AFB1 Chickens

Raman spectroscopy was used to examine the secondary structure of the myofibrillar protein in breast muscle, to determine whether changes in amino acids and their metabolites caused changes in protein structure in breast muscle. Changes in Raman bands of protein chemical groups in proteins shed light on secondary structure changes, such as amide conformation regions, C-C stretching vibrations, tryptophan residues, tyrosyl doublets, and aliphatic amino acids; Amide I (1600–1700 cm^−1^) and Amide III (1200–1350 cm^−1^) are the most useful to determine secondary structure [23]. The secondary structure of the myofibrillar protein changes in breast muscle is exhibited in Figure 5C. There was more content of ɑ-helix structures in the AFB1 group than in the CON group (*p* = 0.385), and it is noted that the AFB1 group had significantly more content of ɑ-helix structures than the AFB1 + TFRD group (*p* = 0.008). Moreover, compared with the TFRD group, the AFB1 group showed an increasing trend of the random coil in the protein secondary structure of myofibrillar, but the difference was not significant (*p* = 0.163). The results suggest that AFB1 can affect the protein secondary structure of myofibrillar in breast muscle by affecting the content of amino acids and their metabolites in breast muscle, which was markedly improved by TFRD treatment.

### 3.7. Effect of TFRD on Antioxidant Activity and Inflammatory Factors

**Figure 6:**
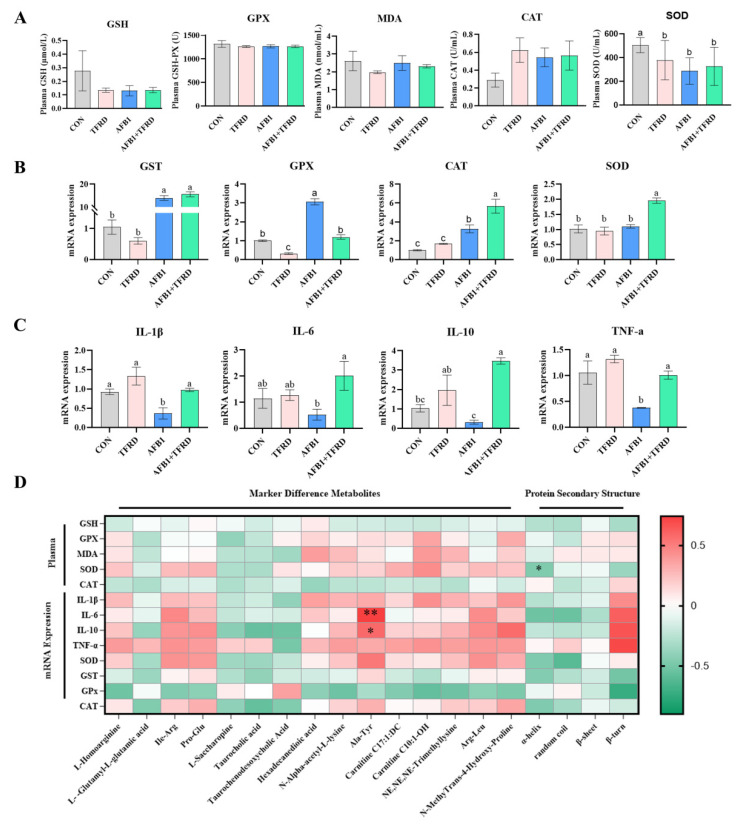
Effect of TFRD on AFB1-induced oxidative stress and inflammatory damage of the breast muscle in broilers. (**A**) Plasma antioxidant enzyme levels, including GSH, GPX, CAT, T-SOD, and MDA. (**B**) The relative mRNA expressions of oxidative stress-related genes *SOD*, *GST*, *GPX*, and *CAT*. (**C**) The relative mRNA expressions of inflammation-related genes *IL-1β*, *IL-6*, *IL-10*, and *TNF-ɑ*. (**D**) Pearson correlation analysis between indicators related to oxidative stress and inflammatory response with the significantly differential metabolites and the protein secondary structure of myofibrillar in breast muscle. Note: The different rectangles are colored based on the Pearson correlation coefficients between meat quality assessment indicators. The intensity of color represents the degree of correlation, green represents positive correlation, red represents negative correlation. The asterisks indicate statistically significant differences and correspond to *p* < 0.05 (*) and *p* < 0.01 (**). Different letters indicate statistically significant differences (*p* < 0.05), *p*-value higher than 0.05 represents not statistically significant.

In this study, the activities or levels of antioxidant parameters (*GSH*, *GPX*, *CAT*, *SOD*, and *MDA*) in plasma and the mRNA levels of antioxidant genes such as SOD, GST, GPX, and CAT in breast muscle were measured by ELISA and RT-qPCR, respectively (Figure 6A). The results revealed that the activity of plasma GSH decreased by 53.1% in the AFB1 group compared to the CON group. Compared to the AFB1 group, the activity of plasma SOD increased by 13.4%, while the activity of plasma MDA decreased by 7.3% in the AFB1 + TFRD group, but the difference was not significant (*p* = 0.635 and *p* = 0.374, respectively). Furthermore, the activity of plasma SOD was found to be significantly lower in the AFB1 group than in the CON group (*p* < 0.01). As shown in Figure 6B, the mRNA expression of *GST*, *GPX*, and *CAT* was significantly increased in the broiler breast muscle of the AFB1 group compared to the CON group (*p* < 0.001, *p* < 0.001, and *p* = 0.006, respectively). It is noted that *SOD* and *CAT* expression levels were significantly elevated in the broiler breast muscle of the AFB1 + TFRD group compared to the AFB1-exposed group (*p* < 0.001 and *p* = 0.004, respectively). These results indicated that TFRD treatment can protect against AFB1-induced changes in the oxidative indicators in broiler breast muscle.

The mRNA expression of *IL-1β*, *IL-6*, *IL-10*, and *TNF-α* in the breast muscle was used as inflammatory response biomarkers. As shown in Figure 6C, the mRNA expression of *IL-1β* and *TNF-ɑ* was dramatically decreased in the AFB1 group compared to the CON group (*p* = 0.024 and *p* = 0.005, respectively). Meanwhile, the expression of mRNA of inflammatory factors IL-1β, IL-6, IL-10, and *TNF-ɑ* was prominently decreased in the broiler breast muscle of the AFB1 group compared to the AFB1 + TFRD group (*p* = 0.017, *p* = 0.021, *p* = 0.001, and *p* = 0.007, respectively). These results indicated that TFRD treatment can protect against AFB1-induced changes in inflammatory response indicators in broiler breast muscle 

Pearson correlation analysis was performed to further investigate the correlation between oxidative stress and the inflammatory response with key differential metabolites and protein secondary structure of myofibrillar (Figure 6D). The results showed that plasma antioxidant enzyme activity and mRNA expression of indicators related to inflammatory response and oxidative stress showed mostly common negative correlation with the protein secondary structure of myofibrillar, plasma SOD activity showed a significant negative correlation with the protein secondary structure of ɑ-helix (r = −0.468 and *p* = 0.028), but the mRNA expression of *IL-6*, *IL-10*, and *TNF-ɑ* showed a significant prominent positive correlation with the protein secondary structure of β-turn (r = 0.620, 0.669 and 0.710, *p* = 0.265, 0.217, and 0.179). Meanwhile, the mRNA expression of *IL-6* and *IL-10* showed a prominent positive correlation with Ala-Tyr (r = 0.743 and 0.581, *p* = 0.006 and 0.048). Together, alterations in chicken meat quality, breast muscle differential metabolites, and secondary structure of the myofibrillar protein may be associated with AFB1-induced oxidative damage and inflammation.

## 4. Discussion

**Figure 7:**
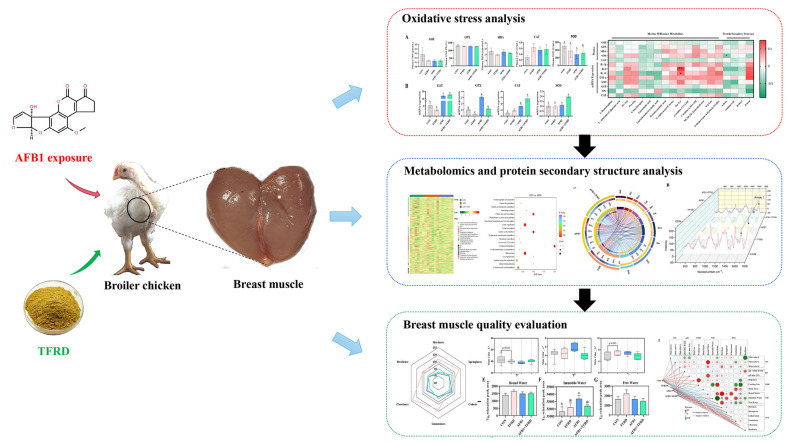
The schematic diagram of total flavonoids of *Rhizoma Drynariae* against meat quality deterioration caused by dietary aflatoxin B1 exposure in chickens.

As the demand for healthier and tastier meat increases, broiler breast muscle continues to gain popularity as a slice of high-quality meat that is high in protein and low in fat [24]. The widespread presence of AFB1 in animal feed not only poses a threat to animal welfare and health, but also has the potential to chicken breast meat, an important animal-derived food, posing a threat to human food health and safety [25,26]. Although the toxicity of AFB1 has received widespread attention, little has been reported on the toxic effects of AFB1 on broiler breast meat quality and how to reduce or detoxify AFB1. In this study, it was confirmed that TFRD addition to the feed could effectively attenuate the alteration of metabolites and myofibrillar protein secondary structure in the breast muscle caused by AFB1 through reducing oxidative damage and inflammatory response, thereby ameliorating AFB1-induced deterioration in chicken meat quality, including the decline in WHC, decrease in TPA indicators and increase in immobile water most abundant in breast muscle (Figure 7).

When it comes to assessing the quality of chicken breast meat, physical characteristics such as the color of the meat, the pH, the WHC, the shear force, and the dynamic distribution of water are usually the first factors to be addressed [27]. The color of the meat is the first factor observed visually by the consumers and predominantly stimulates the desire to purchase chicken meat [28]. Studies have shown that mycotoxins in the diet can negatively impact the meat color of broilers [29]. In the present study, AFB1 exposure significantly increased pH after 24 h and meat color a* values in the breast muscle compared to the CON group, while pH and meat color in broilers supplemented with TFRD maintained the same trend as in the CON group. It is well known that pH value is an essential determinant of meat color, WHC, dynamic distribution of water, and shear force [30].

Furthermore, the evaluation of the WHC of breast meat mainly includes drip loss and cooking loss [19]. In this study, AFB1 significantly increased the drip loss of broiler breast muscle. Meanwhile, the drip loss and cooking loss of broiler breast muscle in the AFB1 + TFRD group were significantly lower than those in the CON and AFB1 groups, which implies that the WHC of broiler breast muscle decreased after AFB1 exposure, while TFRD supplementation significantly improved the WHC of broiler breast muscle. This is consistent with the finding that AFB1 exposure led to a reduction in the water-holding capacity of sheep meat [8]. Bertram et al. [31] indicated an evident relationship between WHC and immobile water’s dynamic distribution as measured by NMR transverse relaxation (T_2_). The present experiment showed that broilers in the AFB1 group had significantly higher T_21_ (immobile water) population and breast muscle ratio than those in the CON group. This phenomenon may be caused by decreased WHC in the breast muscle due to AFB1 exposure, triggering intra-myofibrillar water partially flowing into extra-myofibrillar space and ultimately leading to edema in the broiler breast muscle. Moreover, changes in pH in breast muscle lead to the contraction of myofibrils, and changes in water distribution in the fiber gap lead to differences [32]. Therefore, the results suggest that AFB1 exposure affected the acid removal process in broiler breast muscle, leading to an increase in pH after 24 h, leading to a change in breast muscle meat color and a decrease in the WHC. Optimistically, the deterioration in breast muscle quality caused by AFB1 exposure could be significantly improved by supplementation with the herbal extract TFRD.

Metabonomics, as a new omics analysis technology, has been widely used in relevant animal omics analysis and contributed to animal meat quality analysis [33]. Metabolomic analysis can help to better understand the effects of TFRD on the metabolite composition of AFB1-exposed broiler breast muscle. The levels of metabolites produced by chemical reactions within the animal organism can be considered the ultimate response of the biological system to changes in its genetic composition or its environment [34]. The metabolite profiles of broiler breast muscle were performed in this study. It was revealed that the metabolites in breast muscle altered by AFB1 exposure were mainly concentrated in amino acids, organic acids, GP, nucleotides, FA, and their metabolites. Combined cluster analysis, abundance analysis, and ternary plot analysis revealed that the broiler breast muscle differential metabolites that were more altered between groups were amino acids, bile acids, fatty acids, and their metabolites.

Furthermore, KEGG analysis revealed that the metabolic pathways under AFB1 exposure were mainly enriched in primary bile acid biosynthesis, lysine degradation, linoleic acid metabolism, etc. For TFRD treatment, metabolic pathways were mainly enriched in amino acid metabolism, including lysine degradation and biosynthesis, arginine biosynthesis, etc. Jia et al. [35] similarly found that the identification of differentially expressed amino acids and their metabolites and nucleotides and their metabolites as important factors affecting meat quality by non-targeted metabolomics. Amino acids can provide key nutritional value, and significantly contribute to the taste and flavor of meat [34]. Arginine and lysine are the essential amino acids for the body closely associated with changes in the meat quality of broiler breast muscle. It has been demonstrated that increasing lysine in the diet can increase the final pH of broiler breast muscle and reduce the drip loss of broiler breast muscle [36]. Peng et al. [37] and Zampiga et al. [38] also indicated that increasing the ratio of arginine to lysine can significantly affect the L* and b* values of meat color, pH after 24 h and reduce the rate of white striping, wooden breast in broiler chickens. Thus, metabolomics further characterized the metabolite profile of broiler breast muscle under AFB1 exposure and TFRD intervention, which allowed us to understand the reduction in chicken quality caused by AFB1 exposure in terms of breast muscle metabolite levels.

In the present study, AFB1 exposure resulted in significantly higher isoleucine, arginine, and leucine levels in broiler breast muscle than in the control group. Isoleucine, arginine, and leucine are widely present in poultry as essential amino acids in the body [39]. The amino acids not only act as signaling molecules to regulate the normal metabolism of the body, but also act as flavor substances to influence the flavor of broiler breast muscle [39]. In addition, Pearson correlation analysis found that isoleucine, arginine, and leucine showed a significant positive correlation with meat color a* values and pH after 24 h, which are consistent with the study by Huang et al. [17]. A study by Qu et al. [40] found that heavy metals were also responsible for amino acid disorders in broiler breast muscle, including Arg and Leu. The protein secondary structure of myofibrillar in breast muscle was examined by Raman spectroscopy to investigate whether changes in amino acids and their metabolites in breast muscle cause changes in protein structure. Raman spectroscopy is a fast, non-destructive monitoring method, and the spectra provide quantitative and qualitative data on protein changes for the analysis of the protein secondary structure of myofibrillar. In this experiment, AFB1 exposure was found to cause an increase in ɑ-helix content in broiler breast muscle. Liu et al. [41] found the decrease in pH in fish muscle can cause a significant decrease in ɑ-helix content in fish myosin. Bertram et al. [42] observed the increase in α-helix structures content in muscle was significantly consistent with the increase of myofibril internal water. This is also consistent with our experimental results; AFB1 exposure caused an increase in ɑ-helix of the protein secondary structure of myofibrillar and an increase in immobile water in breast muscle. 

Previous studies have shown that AFB1 exposure can cause oxidative stress and inflammatory damage in the body [43,44]. In addition, the body activates a series of antioxidant reactions to prevent further oxidative damage by increasing the activity of antioxidant enzymes in the body and initiating the degradation of lysosomal enzymes [45]. This experiment showed that AFB1 exposure caused an increase in MDA levels in broiler plasma and mRNA levels for two antioxidant enzymes, *GST* and *GPX*, compared to the *CON* group, suggesting the involvement of oxidative stress in the effect of AFB1 on broiler breast muscle meat quality. According to a previous study, AFB1-glutathione (GSH) conjugation is the major pathway for the detoxification of aflatoxin metabolites [46]. GST plays a major role in regulating the formation of nuclear DNA by the AFB1 adduct, and changes in its activity reduce the formation of the AFB1-DNA adduct (Allameh et al., 2020). In this study, TFRD promoted the expression of GST and thus further promoted the metabolism of AFB1 by regulating the glutathione system [47]. Bhatti et al. demonstrated that dietary exposure to AFB1 alone resulted in degenerative and necrotic changes in the bursa and thymus, ultimately causing a dose-dependent suppression of the immune response in broilers [48]. In the present experiment, the reduced expression of IL-1β, IL-6, IL-10, and TNF-ɑ may be due to the immunosuppression of the broiler caused by AFB1, which in turn reduced the secretion of inflammatory factors. Meanwhile, the addition of TFRD reversed this phenomenon. We examined the TFRD used in this experiment by high-performance liquid chromatography and 97.82% of its component was rutin. Rutin has a significant anti-inflammatory effect in the inflammatory response caused by immune cell transfer [49]. Therefore, we speculate that TFRD may exert a potential protective effect through its main active ingredient, rutin, during the decrease in breast muscle quality induced by AFB1 exposure. The occurrence of oxidative stress and inflammatory responses in the breast muscle is strongly correlated with changes in metabolism in the breast muscle. L-arginine exhibits marked antioxidant properties [50]. Bile acids and cholesterol are closely related to the decomposition and metabolism of fat and changes in their metabolic pathways affect the fat content in tissues [51]. Adipose tissue is an essential source of pro-inflammatory cytokines, and high lipids may up-regulate the production of cytokines [52]. In addition, in correlation analysis alanine and tyrosine showed significant positive correlations with IL-6, IL-10, and pH after 24 h, and GPX showed negative correlations with L-homoarginine, carnitine C10:1-OH and NE, NE, and NE-trimethyl lysine. Collectively, these results suggest that exposure of broilers to AFB1 causes oxidative stress and inflammatory response to the breast muscle, which affects changes in breast muscle metabolite levels and alterations in myofibrillar protein secondary structure, ultimately leading to deterioration in chicken meat quality, including the decline in WHC, decrease in TPA indicators and increase in immobile water most abundant in breast muscle.

## 5. Conclusions

Our findings revealed that AFB1 affects the content of metabolites in broiler breast muscle by inhibiting the secretion of inflammatory factors and disrupting the antioxidant enzyme system, further altering parameters related to breast muscle mass to influence broiler breast muscle quality. In addition, metabolomic analysis screened for several marker differential metabolites associated with oxidative stress, inflammatory response, and breast muscle quality. Optimistically, the addition of TFRD altered the levels of marker differential metabolites in broiler breast muscle through amino acid metabolic pathways, lipid metabolic pathways, and energy metabolic pathways, thereby protecting breast muscle from AFB1-induced meat loss, oxidative stress, and inflammatory response. The present study enables to understand how AFB1 affects broiler breast muscle quality and contributes to the use of TFRD (main ingredient rutin) as a potentially safe and harmless herbal extract in poultry production.

## Data Availability

All data generated or analyzed during this study are included in this published article and its supplementary information files. The data that support the findings of this study are available from the corresponding author [Shu-cheng Huang], upon reasonable request.

## Supplementary Materials

The following supporting information can be downloaded at: https://www.mdpi.com/article/10.3390/antiox12010083/s1, Table S1: The composition of TFRD, Table S2: Assignment of major bands in Raman spectra, Table S3: Primer sequences for quantitative real-time PCR analysis.
