# Supplementary material for: Novel Insights into Total Flavonoids of Rhizoma Drynariae against Meat Quality Deterioration Caused by Dietary Aflatoxin B1 Exposure in Chickens
Source: Antioxidants (Basel). 2022 Dec 30;12(1):83. doi: 10.3390/antiox12010083 (PMC9854432; doi:10.3390/antiox12010083)
Supplement: Supplementary file 1 [file antioxidants-12-00083-s001.zip › antioxidants-2034113-supplementary.pdf]

Table S1: The composition of TFRD

Chemical composition	Content	
	μg/g	%
Rutin	401592	97.8233
Quercetin	7072.626	1.7228
Quercetin 3-glucoside	1119.576	0.2727
Myricetin	192.0882	0.0468
Kaempferol	149.7704	0.0365
Astragalin	148.6356	0.0362
Genistin	79.12365	0.0193
Genistein	47.70714	0.0116
Taxifolin	28.42508	0.0069
Cynaroside	22.18828	0.0054
Formononetin	18.52867	0.0045
Luteolin	18.25931	0.0044
Isovitexin	13.03135	0.0032
Naringenin	9.408299	0.0023
Biochanin A	6.306109	0.0015
Vitexin	4.016406	0.001
Daidzein	2.543361	0.0006
Apigenin	1.512769	0.0004
Liquiritigenin	1.027148	0.0003
Quercitrin	1.00514	0.0002
Glycitein	0.086143	0
Chrysin	0.014538	0

Table S2: Assignment of major bands in Raman spectra

Band assignment	Wavenumber (cm^{-1})[a]
Amide III′ (α-helix)	830, 850
Amide III′ (random coil)	940
Amide III′ (β-sheet)	960
Phenylalanine	1003
Amide III (β-sheet)	1224, 1242
Amide III ((random coil)	1250
Amide III (α-helix)	1265, 1279, 1305
Amide I (α-helix)	1645, 1655
Amide I (random coil)	1665
Amide I (β-sheet)	1667, 1676
Amide I (β-turns**)**	1685

[a] ± 3 - 5 cm^{-1}

Table S3: Primer sequences for quantitative real-time PCR analysis.

Gens	Gene bank ID	Primer sequence (5’-3’)	Products length
IL-1β	XM_015297469.2	F: GACCAAACTGCTGCGGAGGC	154
		R:GAAGGACTGTGAGCGGGTGT	
IL-6	NM_204628.1	F: CTGCCCAAGGTGACGGAG	103
		R:GGTAGGTCTGAAAGGCGAACA	
IL-10	NM_001004414.2	F: TGCCAAGCCCTGTTGCTG	158
		R: TCATCGTCCCTGGATTGAAAA	
TNF-α	XM_040647309.1	F: GGACAGCCTATGCCAACAAGT	168
		R: ACACGACAGCCAAGTCAACG	
SOD	NM_205064.1	F: CCGGCTTGTCTGATGGAGAT	125
		R: TGCATCTTTTGGTCCACCGT	
CAT	NM_001031215.2	F: GGTTCGGTGGGGTTGTCTTT	213
		R: CACCAGTGGTCAAGGCATCT	
GST	NM_001001777.1	F: AGAGTCGAAGCCTGATGCAC	220
		R: CACTCCGCTTATCAGCAAACA	
GPX1	NM_001277853.2	F: ACGGCGCATCTTCCAAAG	73
		R: TGTTCCCCCAACCATTTCTC	
GAPDH	NM-204305.1	F: CCTCTCTGGCAAAGTCCAAG	176
		R: GGTCACGCTCCTGGAAGATA	

Note: F. Forward primer; R. Reverse primer.
